# Modelling the spread of infectious diseases in public transport systems under varying demand patterns and capacity constraints

**DOI:** 10.1038/s41598-025-15237-9

**Published:** 2025-08-15

**Authors:** László Hajdu, Jovan Pavlović, Miklós Krész, András Bóta

**Affiliations:** 1 InnoRenew CoE, UP IAM and UP FAMNIT, University of Primorska, Titov trg 4, 6000 Koper, Slovenia; 2 FAMNIT, University of Primorska, Glagoljaška 8, 6000 Koper, Slovenia; 3 Department of Applied Informatics, University of Szeged, Boldogasszony sgt. 6, Szeged, 6725 Hungary; 4 Embedded Intelligent Systems Lab, Department of Computer Science, Electrical and Space Engineering, Luleå University of Technology, 97187 Luleå, Sweden

**Keywords:** Network algorithms, Public transportation, Epidemic modelling, Civil engineering, Computer science, Epidemiology, Health policy

## Abstract

Understanding the dynamics of passenger interactions and their epidemiological impact across public transportation systems is crucial for both service efficiency and public health. High passenger density and close physical proximity have been shown to accelerate the spread of infectious diseases. During the COVID-19 pandemic, many public transportation companies took measures to slow down and minimize the spread of the disease. One of these measures was introducing spacing and capacity constraints on public transit vehicles. Our objective is to explore the effects of changes in demand and transportation measures from an epidemiological point of view, offering alternative measures to public transportation companies to keep the system operational while minimizing the epidemiological risk as much as possible. Our findings show that restricting vehicle capacity can significantly reduce the spread of infections, while demand-related measures have an even stronger effect. Combining these approaches offers the best solutions for balancing public health and operability.

## Introduction

Recent years have highlighted the importance of understanding the dynamics of epidemiological transmission in public spaces, particularly within public transportation systems. Due to the large number of interacting and co-traveling passengers, these environments can act as potential accelerators and hotspots for the spread of different viruses, especially during a global pandemic. During the COVID-19 outbreak, physical distancing was one of the most common non-pharmaceutical measures used to prevent the spread of the disease^1^. A good overview of COVID-19-related restrictions can be found here^2,3^. While these measures help mitigate transmission, they often lead to efficiency and operational problems, redirect passengers to other travel options, or prevent them from being able to travel in the city, thereby causing disruptions and potentially increasing epidemiological risks in other environments. Therefore, investigating and understanding how the combination of fluctuations in passenger behavior and restrictive measures influence disease transmission is vital to providing an innovative and safe solution for society.

Agent-based models provide us with reliable solutions to approximate the behavior of public transportation users in the city. The structure of the public transportation system, including schedules, vehicle trips, or stations, defines a unique restricted environment where agents/passengers use the services, move between stations, and interact with each other in vehicles^4^. The other dimension of the system is demand, which defines the patterns and timing of passengers’ movement within the city to meet their objectives. Public transportation systems are characterized by numerous parameters, making them complex, sensitive, and difficult to predict, particularly when assessing the epidemiological effects of these parameters.

During a pandemic, various aspects of the system can change, including its structure (due to restrictions) and demand (due to altered passenger needs)^5,6^. Consequently, both structure and demand must be analyzed together to understand the overall behavior of the system.

A wide range of epidemiological models exists, from traditional compartmental models^7^ to the Independent Cascade^8^, or Linear Threshold^9^ models. Compartmental models, such as SI, SIR, or SEIR models, are commonly used to simulate the spread of diseases and approximate outbreak scenarios. Originally, compartmental models were introduced to simulate the spread of diseases in a homogeneous space using differential equations; however, the concept has since been extended to networks^8,10,11^. Many studies use agent-based simulations combined with compartmental models to simulate the spread of infectious diseases and information on contact networks, helping to predict and evaluate intervention strategies^11,12^. Lastly, simulating epidemiological models on contact networks requires transmission risk values between the network nodes. Multiple models have been proposed to estimate these values based on real-life observations^13–15^.

Smart card data^16^ can capture detailed passenger contact patterns that are essential for developing epidemic surveillance and containment strategies. Creating accurate real-world contact networks from these data poses challenges such as computational complexity and privacy issues^17–19^. However, a more precise interaction network allows us to model the epidemiological spread more realistically^20–22^.

The literature offers a wide range of papers that provide an overview of epidemic modeling on public transportation^23–25^. Most existing approaches focus on analyzing historical use cases alongside previously implemented restriction scenarios^26–28^. However, there are notable gaps in the literature regarding simulation and evaluation of how different measures impact existing public transportation systems from an epidemiological point of view.

This paper aims to study the behavior of the San Francisco Bay Area public transportation system during multiple simulated epidemic outbreak scenarios and potential authority-imposed restrictions. Specifically, we seek to measure changes in infection rates and network operability to find a balance between preventive measures and maintaining the public transportation system’s ability to allow passengers to reach their destination. Our approach follows the methodology of^29,30^ by generating passenger contact networks that reflect changes in public transit demand and vehicle capacity restrictions. Infection processes are simulated on these networks to evaluate how different demand- and restriction-related changes affect the spread of the disease and the operability of the system.

We acknowledge that a large-scale epidemic outbreak has wide-ranging impact on society, and public authorities may introduce a large variety of preventative or mitigating measures to combat spreading. Modelling vehicle capacity limitations and changes in demand represent only a subset of possible scenarios. Due to limitations in data availability, we only consider these two changes in this study. The following sections provide a brief literature review, introduce the route choice model, outline the contact network structure, and the epidemiological modeling algorithm.

## Literature overview

This section is a review of selected studies on modeling epidemic spread within public transportation networks.

Multiple studies have focused on evaluating the effectiveness of interventions aimed at reducing transmission risk. In^31^, the authors studied social distancing interventions in public transportation across the U.S.A. and Canada. They highlighted measures such as limiting seating capacity, reducing bus and railcar occupancy, and using signs or physical barriers to enforce distancing. While improving safety, these measures resulted in higher operating expenses and significant loss in income due to lower fares. The research paper^32^ evaluated COVID-19 safety measures implemented by Polish public transport agencies, comparing residents’ safety preferences with strategies adopted by service providers. Limiting passenger numbers to 50% of seating capacity was a key intervention, which was later revised to include standing capacity as well. Similarly, another study^33^ explored infection control strategies in Bangkok, including mandatory face masks, social distancing, and passenger capacity caps. The study focused on long-lasting behavioral shifts, with travelers either avoiding traveling altogether or switching to private vehicles. This highlights how crucial it is to balance passenger demand and public health initiatives. The authors in^34^ employed an adapted Wells-Riley model to assess the risk of COVID-19 infection on a bus route in Houston, Texas, considering multiple scenarios including ventilation, social distancing, mask use, vaccination, and duration of exposure. The results indicated that decreasing bus capacity decreases the risk of infection, while the increased operational costs of social distancing could be reduced by implementing a proposed “split route strategy.”

Large-scale simulations have been used to evaluate the risks of infection and model the effectiveness of interventions. For example, the study in^35^ focused on the transit system in Seoul, South Korea, using an agent-based modeling approach similar to ours. This study incorporated smart card data with origin-destination pairs, a transit assignment model, and a SEIR simulation on a passenger contact network. However, it did not explicitly model capacity constraints but instead considered different congestion scenarios without accounting for varying demand levels. The findings indicated that imposing social distancing reduces the risk of infection, although not as effectively as implementing mask-wearing alone or combining the two policies. In^36^, the authors used smart card data from Singapore’s bus system to create a time-varying Public Transit Encounter Network (PEN) together with a SEIR model to model the spread of infectious diseases. They assessed strategies such as bus route closures, physical distance, and passenger load limits. Reducing vehicle capacity was found to marginally slow the spread of infection while preserving urban mobility. Although less effective than route closures, limiting bus capacity balances infection control while preserving transportation accessibility. One study^37^ integrated a mathematical optimization method with an infection risk model to formulate operational strategies for public transit during a pandemic, concentrating on vehicle capacity, frequency of staff testing, and service scheduling. Numerical simulations utilizing actual transit data indicated that reduced vehicle capacity and optimal profit are connected to passengers’ expectations of close contact, while larger buses contributed to maintaining safety protocols. The research concluded that transit operators should prioritize routes that are in high demand, that have significant penalties for unserved passengers, or routes that have higher exposure risks when allocating resources. In addition, the study in^38^ proposed an agent-based simulation framework to assess COVID-19 transmission on U.S. buses, by modeling passenger behavior and virus movement based on seating, airflow, and proximity. Three scenarios were assessed by simulation: no limitations, mandatory mask use , and a combination of masks, improved ventilation, and half-capacity seating. Results indicated that the combination of masks and half-capacity seating nearly eliminated the risk of infection. However, the study did not consider varying passenger demand alongside capacity restrictions.

Kilani et al.^39^ investigated the modeling of virus propagation on a regional scale (Northern France) by evaluating the impact of public health interventions such as mask mandates and lockdowns. Their study simulated interactions across a range of activities, including work, leisure, and education. The authors used an activity-based modeling technique to define infection probabilities for various activities and means of transportation, with elementary schools being identified as a major source of transmission. While their approach provides a broad strategic overview of intervention scenarios, unlike our study, it does not go into great detail on public transit, which is just one of several factors that was examined. In contrast, our study adopts a city-level and transportation-specific approach, focusing primarily on the public transportation system, to examine the demand-capacity tradeoff and vehicle trip-based interactions.

Overview papers related to this topic can be found in the following articles^40–42^. Another study^43^ employed agent-based modeling to simulate the transmission of respiratory infectious diseases in public transportation systems, focusing on a 24-seat train wagon and a 49-seat public utility bus. The model accounted for passenger interactions, seating arrangements, and movement patterns to evaluate infection dynamics under various conditions. Capacity reductions were modeled with crowd density ranging from 10 to 100%. The findings showed that reducing vehicle capacity and increasing physical distance between passengers significantly reduced the risk of infection. For instance, maintaining passenger capacity below 50% with enforced social distancing substantially reduced the infection rate. In scenarios with 10% capacity and stringent protection measures, infections were nearly eliminated.

## Methods

In this paper, we use a schedule-based transit assignment model to simulate highly probable passenger paths on the public transport network of the San Francisco Bay Area using real-life data. The model provides accurate travel time estimates and transfer waiting times. After simulating the behavior of passengers in the city, we constructed contact networks, computed properties of these networks, and analyzed the operability, defined as the ability of the system to deliver passengers to their destinations, in each proposed scenario. Outbreak processes were then simulated using the discrete SIR model to assess infection spread and identify the effects of demand changes and restriction-related scenarios. The next section introduces the route choice model we used for simulating passenger behavior.

### Transit assignment model

We used the FAST-TrIPs^44^, a schedule-based transit assignment model, for this work. This model first generates hyperpaths for each passenger based on a defined logit route choice model and then assigns individual passengers to their path choices using hyperpath probabilities. Then, it simulates passenger journeys using a mesoscopic transit passenger simulation module. As a dynamic transit assignment model, FAST-TrIPs assigns passengers to vehicles while accounting for the schedule and updating passenger flows. If some passengers failed to board vehicles, their path choices were re-evaluated. Additionally, boarding, alighting, and crowding can affect vehicle dwell times. The model updates costs and probabilities based on actual vehicle trip times and identifies new paths for unassigned passengers. This process is repeated for a prespecified number of iterations or until a convergence criterion is met. Due to the unavailability of a calibrated transit route choice model, we used the model from^30,45^. The route choice utility function is the following:

$$\begin{aligned} u = t_{IV} + 1.77t_{WT} + 3.93t_{WK} + 47.73X_{TR} \end{aligned}$$

where $u, t_{IV}, t_{WT}, t_{WK}$ and $X_{TR}$ represent path utility, in-vehicle time, waiting time, walking time, and number of transfers in a transit path, respectively. To understand the underlying algorithm, FAST-TrIPs operates on a network of nodes that represent stops. Each vehicle trip belonging to a specific transit route is linked to the stop it serves, along with the corresponding arrival and departure times. Each stop contains information about the vehicle trips serving it, and transfer links connect stops where passengers can switch vehicles. This structure allows for precise modelng of vehicle movements and passenger transfers across the transit network.

The core of FAST-TrIPs is the Transit Hyperpath Algorithm, which constructs a subnetwork of probable transit routes and assigns probabilities to these routes using a logit route choice model. The algorithm calculates hyperpaths by considering user-preferred arrival times and waiting time windows, simulating passenger journeys with a focus on real-time decision-making and path selection. Passenger movements are then modeled using a pre-estimated route choice model incorporating in-vehicle time, waiting time, walking time (for access and transfers), and transfer penalties.

The model output contains the individual passenger trajectories, including walking from the origin to the bus stop, boarding and changing vehicles, walking between vehicle trips, and the final walk to the destination. The simulation represents passenger movements on the public transportation system for one full day. The next section introduces the structure of the contact network, focusing on the connection between the output of the FAST-TrIPs model and the network structure.

### Contact network

As discussed in the previous section, the output of the FAST-TrIPs model describes the trajectories together with the exact vehicle trips, walks, and timestamps for each passenger using the transit system. The output can be further processed, creating a network structure called contact network. The objective of this network is to describe passenger interactions on vehicle trips. More precisely, let $G = (V,E)$ be an undirected network, where each passenger using the public transportation system is considered a node; therefore, *V* denotes the set of traveling passengers, while *E* represents the co-traveling set among passenger pairs as a set of edges connecting passengers with sharing a vehicle trip for a positive time. The concept of a vehicle trip refers to a specific route with a particular departure time executed by a single vehicle.

*VT* represents the set of vehicle trips. For a pair of passengers traveling together on vehicle trip $j\in VT$, the corresponding edge $e_j$ is extended with $e^{t_s}_{j}$ and $e^{t_e}_{j}$ denoting the start and end time of the shared part of their travel on trip *j*. Using $e^{t_s}_{j}$ and $e^{t_e}_{v}$, the elapsed time during which the passengers were in contact on the actual vehicle trip can also be calculated. Therefore, edges also describe the strength of the connection between passengers, which can later be used to define a probability that expresses the likelihood of transmitting infection through the connection. In summary, the contact network defines the detailed structure of pairwise passenger connections.

**Fig. 1:**
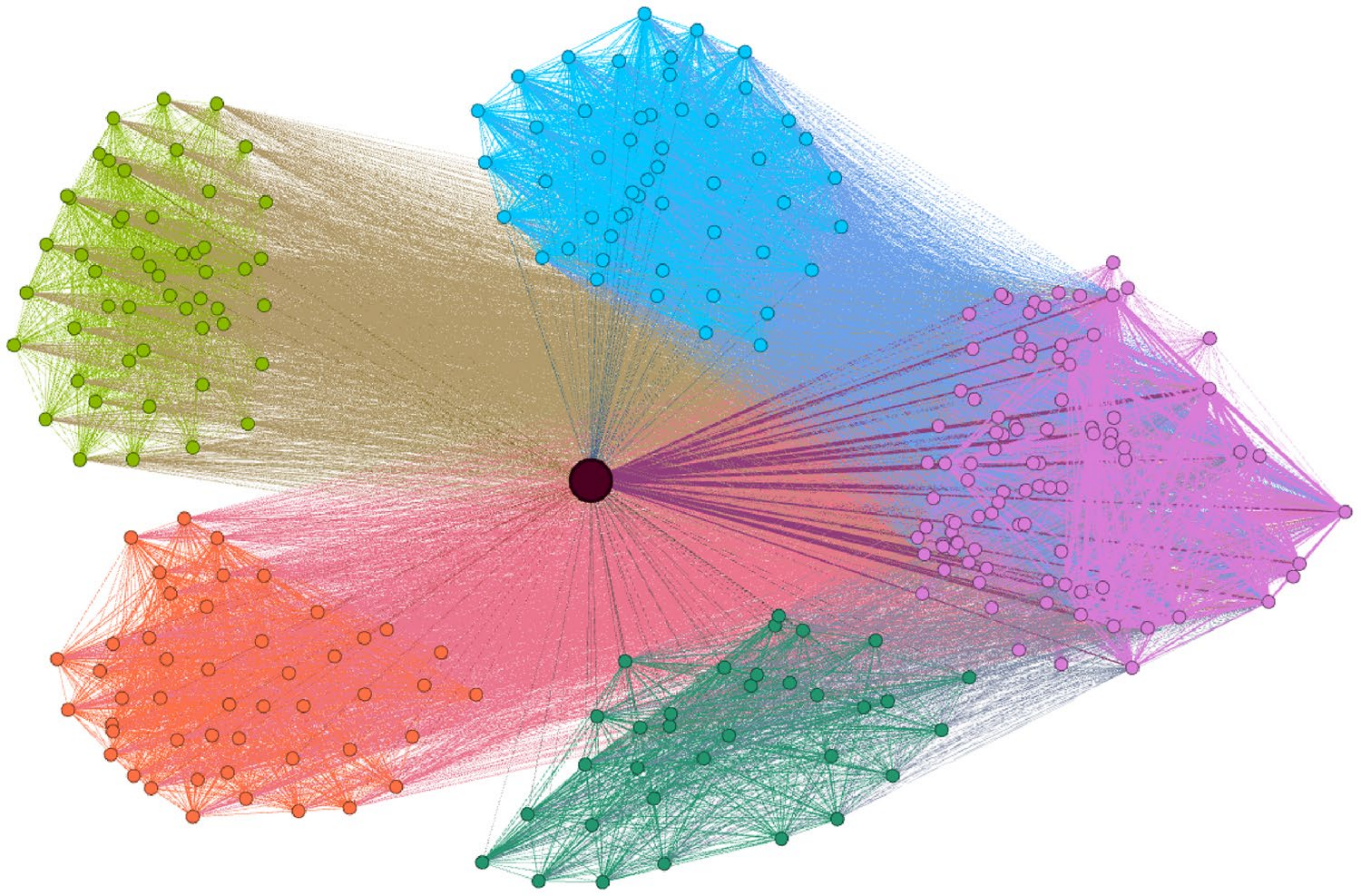
Neighbourhood of the highest-degree passenger in the contact network. Each node represents a passenger, while each edge is a connection between them on a vehicle trip they used together.

Figure 1 shows a subgraph of the contact network around the highest-degree passenger. Different colors correspond to different vehicle trips. It can be seen that the passenger with the highest degree shares many different vehicle trips with different strongly connected groups on the transit system. In the following section, we introduce the epidemiological model used to simulate the spread of a virus using the contact network in the case of an outbreak.

### Epidemiological modeling

We use the susceptible-infected-removed (SIR) compartmental model to represent the different epidemiological scenarios and changes in the transit system. Before we apply the SIR model to the contact network *G*(*V*, *E*), we assign a transmission probability $w_e \in [0,1]$ to each edge $e \in E$, representing the likelihood of infection spreading between the connected nodes. According to the definition of the SIR model, nodes in the network can exist in one of three states: susceptible (S), infected (I), or removed (R). Nodes also have behaviors: a susceptible node can become infected; a removed node was infected once but is no longer contagious, while the infected state corresponds to the case when the node is currently spreading infection to its neighbors.

At the beginning of each simulation, we choose an initial seed set $A_0$ that defines the group of initially infected passengers. The initial seed set can be selected through preliminary knowledge, simulating a historical epidemiological scenario, knowing the origin vehicle trip, and following the infection route through the different parts of the system. On the other hand, the initial seed set can also be chosen randomly, focusing on the structural behavior of the network and introducing an additional stochastic parameter to the modeling. In this study, we randomly selected the initial seed set to explore the structural properties of the public transit system.

During each simulation iteration, infected nodes attempt to transmit the infection to susceptible neighbors based on the edge transmission probability $w_e$. If transmission is successful, the susceptible neighbor transforms to the infected state in the following iteration, continuing the spread of the infection. The infected nodes usually remain in this state for $\tau _i$ iterations before transitioning to the removed state.

The model behaves stochastically, which means that outcomes vary across simulations. By running the simulation *k* times, we can estimate the probability of each node becoming infected by calculating the proportion of simulations in which the node becomes infected.

In this application, each iteration corresponds to a single day, and nodes are assumed to remain infected for five days. As a result, we did not transition passengers to the removed state throughout our simulations. In this way, since each passenger gets the infection only once, our model is identical to the SI variant of the SIR model. Algorithm 1 summarizes the simulation process. In the following section, the calculation of the edge probabilities in the network is presented.

**Algorithm 1:**
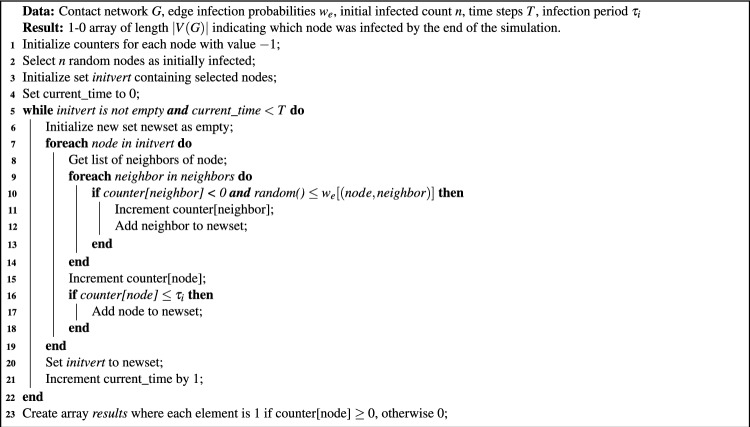
SIR model simulation.

In order to assign realistic transmission probabilities to edges in the contact network, the function introduced in^13^ was used. Probabilities were assigned to edges in the contact network using the following formula:

$$\begin{aligned} w_e = \min \left( P_{max}, \frac{P_{max}}{D_{max}} \cdot D_e\right) \end{aligned}$$

Where $P_{max}$ is the maximum possible transmission probability, $D_{max}$ represents the duration threshold after which an edge is assigned the maximum probability, and $D_e$ is the contact duration associated with edge $e$. The following section introduces the Input data and the various reduced demand and vehicle capacity scenarios.

## Input data

The route choice model works with multiple input files that define the public transportation system and demand. The General Transit Feed Specification (GTFS and GTFS PLUS)^46^ is a standardized format for specifying transit schedules and geographical information. On the other hand, the transit demand data contains information about the trips individual passengers want to make throughout the day, including trip origins, destinations, and preferred arrival times. Additionally, path weights associated with in-vehicle time, waiting time, walking time, and transfer penalties must be specified as input. In summary, GTFS PLUS defines the public transportation network of the city, while demand data describes the different travel needs of passengers.

In our study, we used the General Transit Feed Specification of the San Francisco Bay Area in California from 2017. The transportation system includes 854 routes for bus, heavy and light rail, and ferry traffic. Altogether, 36,058 trips serve 6181 stops over a 24-h weekday.

Since real-world demand data was inaccessible, we utilized data generated for 2017 by the SF-CHAMP travel forecasting tool^47^. SF-CHAMP is an activity-based transportation demand model developed by the San Francisco County Transportation Authority to simulate individual and household travel behavior. By utilizing observed travel patterns, detailed transportation system data, and socioeconomic inputs, SF-CHAMP generates travel demand estimates that are very sensitive to passengers’ behavior. This offers a more accurate and nuanced representation of demand compared to traditional trip-based models.

**Fig. 2:**
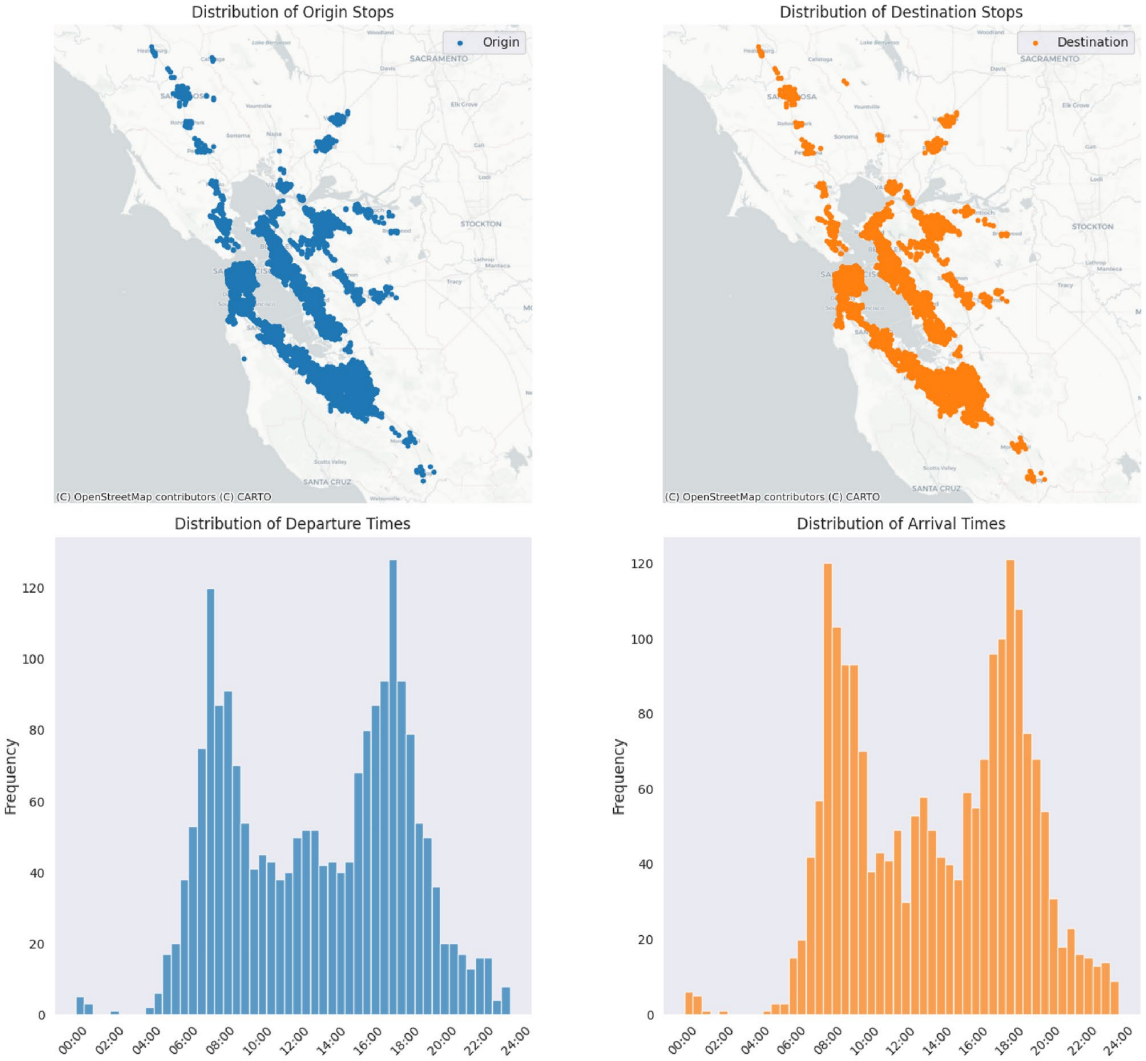
Properties of the original demand data sample. The upper two figures show the spatial distribution of the origin and destinations of the passenger tours, while the lower two show the temporal distribution of the tours. The upper two figures were created with Python version 3.11.10 using library contextily (v. 1.6.2).

The input configuration we used is publicly available on the link provided in the data availability section at the end of the paper. While the provided sample is small, it exhibits desirable characteristics, such as a realistic distribution of passenger departure times that reflects typical weekday commute patterns and a spatial distribution of origin-destination pairs that spans the entire transit network (Fig. 2). Based on these qualities, we extracted a larger sample consisting of 117,500 passenger trips to serve as the demand data in our baseline scenario. To create the final set of passenger trips, we used sampling with replacement, where the individual elements are passenger tours consisting of all the trips made by a single passenger throughout the day. This way, we eliminated the possibility of picking a non-feasible trip configuration while keeping the properties of a realistic demand as can be seen in Fig. 2.

Although the transportation system in the study area has the capacity to serve millions of passenger trips daily, computational limitations led to the use of a smaller dataset. This approach enabled us to preserve the essential elements of realistic trip patterns while maintaining computational viability. Our goal was to demonstrate the new methodology as a ”proof of concept” and the scale of this case study preserves the relevant characteristics of the problem.

**Fig. 3:**
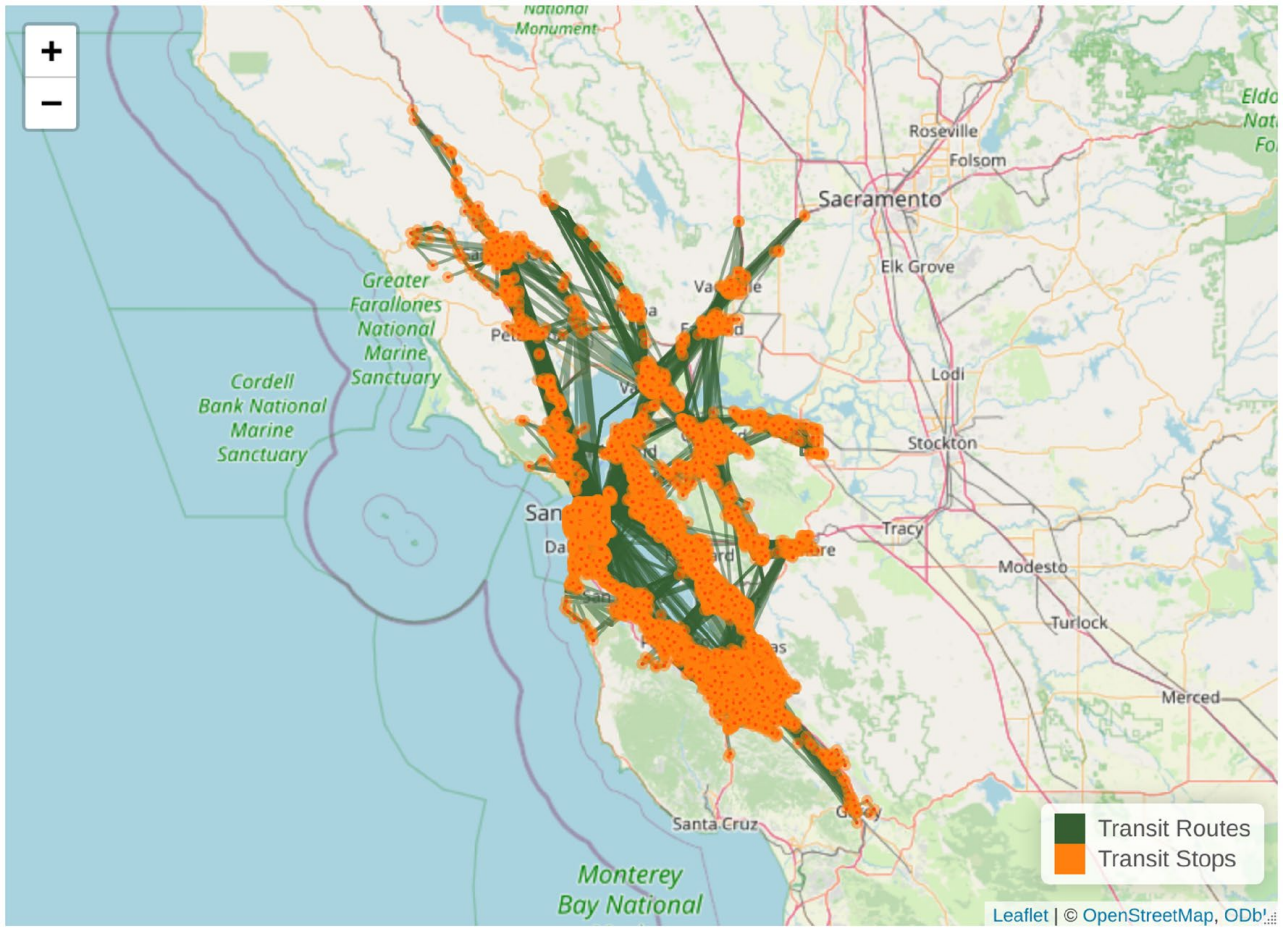
Visualization of transit system. Figure created with R version 4.1.2 https://cran.r-project.org/bin/windows/base/old/4.1.2/, using leaflet 2.2.2 https://cran.r-project.org/package=leaflet. The base tile layer was downloaded from OpenStreetMap (CC BY-SA 2.0; https://www.openstreetmap.org/copyright).

As path weights, we used data from the previous study^45^, corresponding to the Austin, Texas region. Figure 3 shows the public transit system, including the routes and stops in the San Francisco Bay Area, California.

**Fig. 4:**
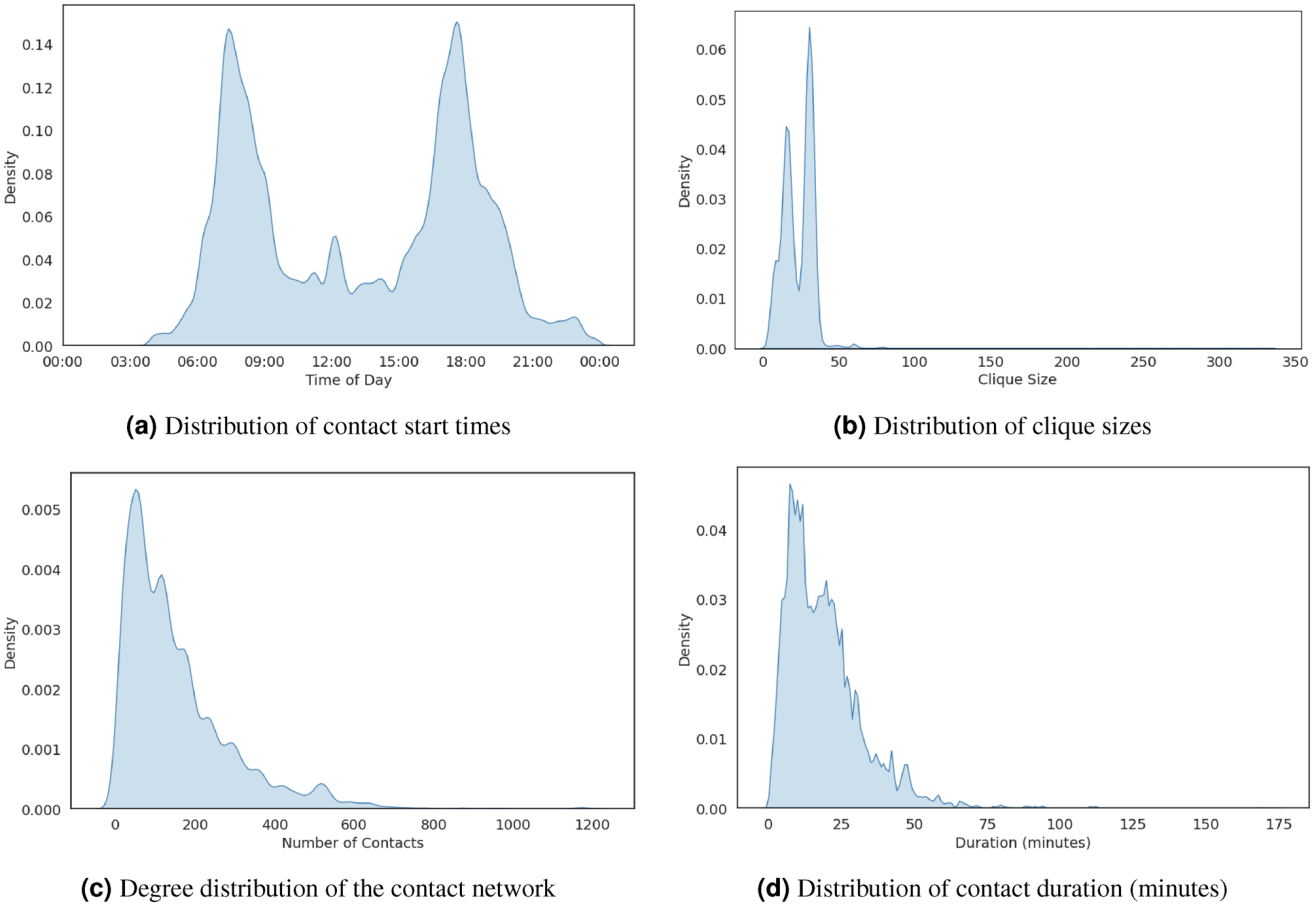
The distribution of (**a**) contact start times, (**b**) clique sizes, (**c**) the number of individual passenger connections, and (**d**) contact durations.

For the baseline demand generated by the SF-CHAMP tool, the contact network constructed from the FAST-TrIPs outputs consisted of 48,546 passenger nodes and 3,756,340 contact links. Figure 4a shows the network’s density plot of contact start times. It can be seen that traffic peaks around 7 a.m. and 5 p.m., reflecting a typical weekday commute pattern where people use public transportation to go to work in the morning and travel home in the late afternoon. The network forms a fully connected subgraph or clique when passengers travel together between stations in the same vehicle. The distribution size of these patterns can be seen in Fig. 4b.

The distribution is heavily right-skewed, with most cliques having a relatively small size (below 30). There are two notable peaks, one between clique sizes 0 and 20 and another around 30. Significantly, few cliques extend beyond size 50. This suggests that significant passenger interactions are typically localized, with occasional larger groupings that may occur on high-capacity vehicles (such as trains). The network’s degree distribution, which follows a skewed power law with an average degree of 154 and a maximum degree of 1193, is shown in Fig. 4c. In contrast, Fig. 4d displays the distribution of contact durations. Most contacts last several minutes, with an average contact duration of 19 min and 13 s.

### Design of transit scenarios

To simulate a variety of potential behaviors during an epidemic outbreak, we defined multiple scenarios that changed the original public transit network and demand. The baseline simulation used the unchanged transit demand and vehicle capacities. Our demand contained 117,500 passenger trips made by 52,816 passengers. To define realistic scenarios regarding the demand changes, we used the Google Mobility Report for California from September 2022. Based on the report, the average decrease in mobility trends for public transportation hubs (such as subways, buses, train stations, etc.) was $-33\%$ compared to the baseline, which means during a COVID peak, $33\%$ fewer passengers used the transit system than before. However, mobility trends fluctuated throughout the month. Therefore, we created four new demand scenarios excluding $17\%$, $33.5\%$, $41\%$, and $50\%$ of passengers from the original demand, along with all their associated trips. To model social distancing measures, we also decreased the capacity of each vehicle in the public transit system, reducing the capacities in the GTFS PLUS descriptions by $10\%$, $20\%$, $30\%$, and $50\%$. This resulted in four different transit supply inputs. Each pair of demand and supply files represents a single use case, resulting in a total of 20 different scenarios (21 with the baseline scenario), highlighting different outcomes with decreased demand and possible epidemiological measures. The following table introduces the different scenarios in detail.

The study^48^ described a model for estimating the risk of COVID-19 infection in public transportation, taking into consideration factors such as exposure time, mask efficiency, ventilation rate, and distance. It demonstrates that the risk of infection lowers as the number of passengers in the vehicle decreases, with the risk heavily influenced by the arrangement of passengers within the vehicle. Another study^49^ estimated the transmission probability of COVID-19 using infection data from the Diamond Princess Cruise Ship, Monte Carlo simulations, and an improved Wells-Riley model. This study calculated the reproductive number *R*, which represents the average number of secondary infections caused by a primary infected individual during their infectious period. The value of *R* for a 48-seat bus during a 2-hour ride was estimated to be 7.48. It was also found that *R* decreases almost linearly as the number of passengers decreases; for instance, when capacity is reduced to 24 passengers, *R* is estimated to be 6.57.

In the epidemiological scenarios we designed, we also aimed to incorporate changes in transmission probability due to reduction in vehicle capacity. Due to the lack of detailed information regarding parameters specific to transit vehicles, such as ventilation rates and seating arrangements, and variation in passenger numbers across different vehicles and times, and given the focus of our study on virus transmission in buses, we opted to use simple heuristics in our approach. Based on the reproductive numbers estimated in^49^ for a 2-hour ride in a 48-seat bus under various capacity constraints, we divided these values by the total number of seats in an average bus to estimate the transmission probability. Consequently, we set the value of $P_{max}$ to 0.163 for the scenario with unchanged vehicle capacity and to 0.16, 0.158, 0.156, and 0.14 for scenarios with capacity reductions of 10%, 20%, 30%, and 50%, respectively. The exact values are in Table 1.

**Table 1:** Infection probability under different capacity reductions.

Capacity	Infection probability ranges on the edges (%)
100% capacity	0–16.3
90% capacity	0–16.0
80% capacity	0–15.8
70% capacity	0–15.6
50% capacity	0–14

## Results and discussion

Our results focus on the effects of reducing demand and vehicle capacity on passenger interactions. The first part highlights the main changes in passenger behavior and differences in passenger travel patterns. The second part focuses on the epidemiological effects to identify which scenario had the most significant effect on the number of infected passengers. Finally, at the end of the result section, we identify the critical parts of the system from an epidemiological point of view.

### Effects of reduced demand and vehicle capacity

**Fig. 5:**
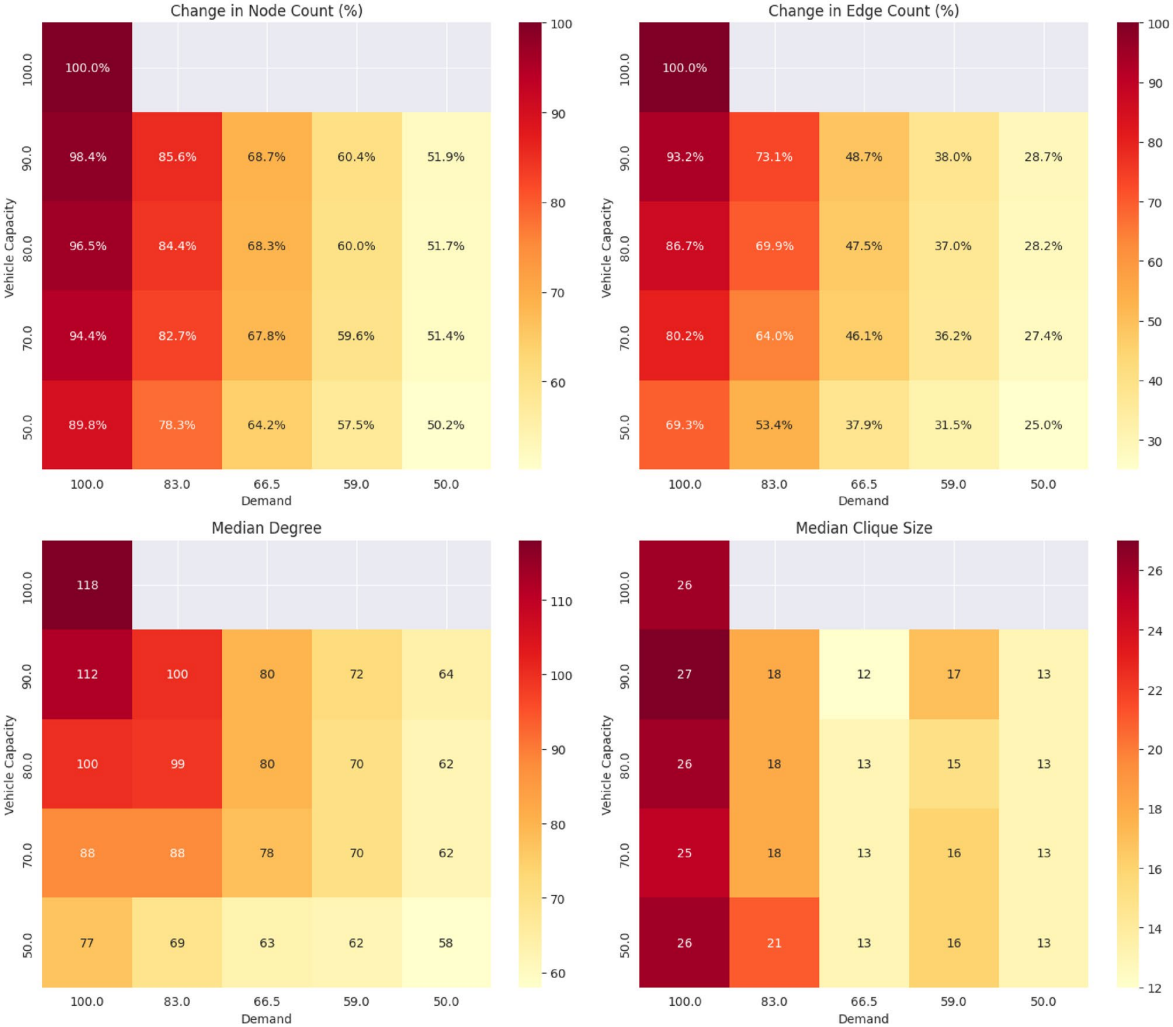
Heatmaps highlighting changes in key metrics in the contact network across different scenarios; The contact network in scenarios with maximal demand and no vehicle capacity restrictions consists of 48,546 nodes and 3,756,340 edges.

As discussed in previous sections, the contact network describes the fine structure of passenger co-traveling relationships during the day. Figure 5 summarizes the changes in key network metrics of the contact network under different demand and vehicle capacity restrictions. These metrics include the number of passengers (nodes), the number of connections (edges), as well as median degree (co-travel connections of individual passengers). If a vehicle travels between stations A and B, passengers sharing the trip will form a clique (fully connected subgraph) in the network. Therefore, metrics also include median clique sizes describing the size of the co-traveling groups between stations. The results shown in the table suggest that capacity restrictions and demand-related changes effectively disrupt the formation of large high-risk clusters, potentially reducing the risk associated with infectious passengers within the transit network.

The results show a significant decrease in the overall size of the passenger contact network (as shown by the number of nodes and edges) across the different scenarios. These changes highlight the impact of the demand and vehicle capacity changes on the network structure.

The restrictions also influenced the degree distribution of the contact network. As the degree of a given node represents the number of direct contacts a passenger has during the day, the risk of exposure to infectious diseases is reduced as demand and capacities decrease. At 100% demand, the median number of passenger connections was 118. Across the different scenarios, this number dropped to 58.

Diseases tend to spread more quickly inside a community or a strongly connected cluster due to the higher number of connections among passengers. Therefore, as passenger groups grow and become more interconnected in the network, they promote the spreading process, working as super spreaders during epidemiological simulation. Consequently, clique sizes and clique size distributions are expected to strongly correlate with the final epidemiological modeling results.

### Demand serving functionality of the transit system

The structure of the transit system does not always match the given structure of demand due to limitations in infrastructure. A natural example is when too many passengers attempt to reach a destination simultaneously, but only a limited number of vehicle trips are available to serve that station. If the system cannot handle individual passengers due to the infeasibility of their demand, they cannot reach their destination, and the route choice model leaves them out of the simulation and the contact network simultaneously. Therefore, for each scenario, a small portion of passengers cannot satisfy their demand due to restrictions in vehicle capacity. This enables the identification of critical parts of the system from an operability point of view, designating vehicle trips that are sensitive to reduced capacities, leaving passengers without transit options. This information can also be used to evaluate the system’s capability to serve passengers. As seen in Table 2, the number of passengers who are unable to reach their destinations increases as restrictions in vehicle capacity are introduced. Consequently, reducing demand naturally reduces the load on sensitive vehicle systems, facilitating system efficiency.

**Table 2:** Percentage of passengers unable to reach their destination during the simulation. In the scenario with full demand and no capacity restrictions, 8% of passengers fail to reach their destination.

	90% capacity	80% capacity	70% capacity	50% capacity
100% demand	9.6%	11.3%	13.2%	17.4%
83% demand	7.4%	8.8%	10.6%	15.3%
66.5% demand	6%	6.6%	7.3%	12.2%
59% demand	5.4%	6%	6.6%	10%
50% demand	5%	5.2%	5.8%	8%

Under normal operating conditions, with 100% demand and unrestricted vehicle capacity, 4270 passengers failed to reach their destination. This serves as a baseline for comparing the effects of different scenarios. Table 2 shows that as capacity restrictions are introduced, the number of passengers with infeasible demand grows substantially. The trend worsens as capacity restrictions become more strict, reaching as many as 9203 stranded passengers at the end of the restriction range. This means that introducing only transit system-related restrictions significantly damages operability. However, even a slight decrease in demand helps ease the load on critical components, helping the system serve a larger portion of passenger demand.

The results demonstrate the necessity of balance between capacity restrictions and demand management. Combining different demands and reducing vehicle capacity can help maintain operability while protecting passengers in case of an outbreak.

### Epidemiological spread analysis

This section is an overview of the epidemiological simulation results, which focus on the global rate of infected passengers and the number of highly endangered individuals. We also provide these values as a ratio compared to the network size in each scenario to compare the real epidemiological effects of restrictions.

At the beginning of each simulation process, we started with 100 infected passengers ($|A_0| =100$), selected randomly from the network, and then we simulated 5 iterations representing an infection period of 5 days. We ran the epidemic spreading process $k = 100,000$ times to estimate the probability of infection. As discussed previously, we selected $\tau _i$ so that no nodes transitioned to the removed state, remaining infectious throughout the simulation.

**Fig. 6:**
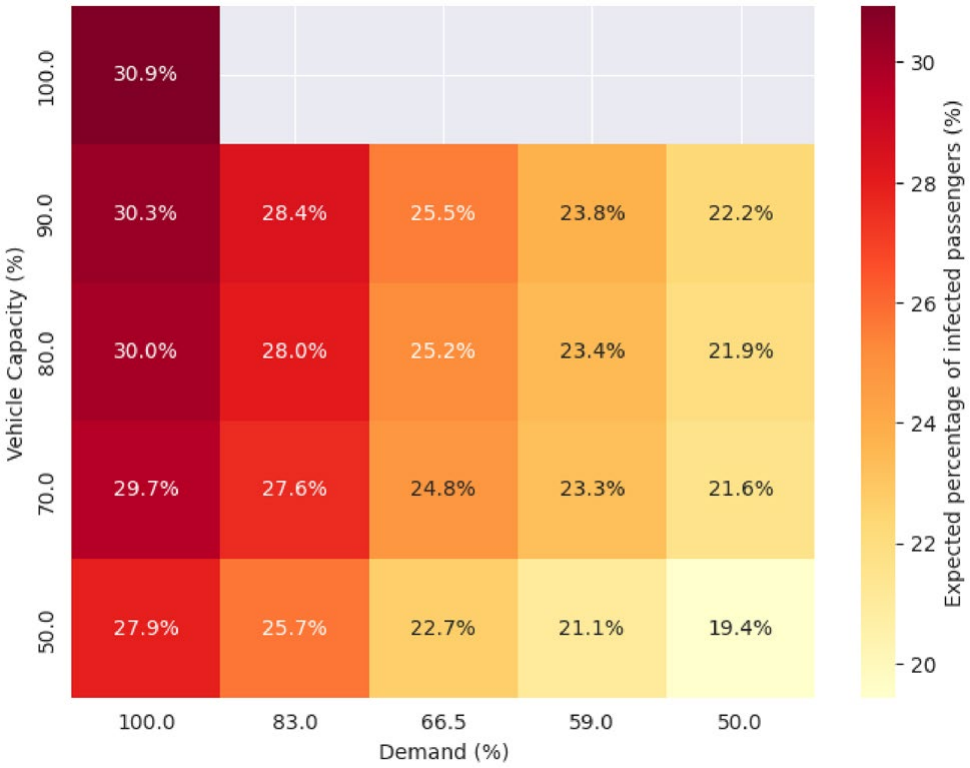
Infection risk associated with each scenario (demand and vehicle capacity).

The epidemiological simulation shows that reduced demand and vehicle capacities affect the global infection rate of the contact network. Figure 6 presents the expected percentage of passengers who are infected at the end of the simulation.

The baseline scenario that did not have reduced demand or restricted capacities shows an infection rate of 30.9%. As the capacity restriction becomes more strict, global infection slightly declines, reducing to 27.9%. A similar but more pronounced trend can be observed for lower demand levels. For example, a 50% reduction in demand results in 8.5% decrease in the risk of infection in the scenario where vehicle capacity is set to 90%. Results show that the reduction in infection rate is more noticeable in the case of demand change than in restricted capacities. However, implementing capacity restrictions, even without significantly reducing passenger demand, can reduce infection rates within the transit network. Furthermore, the combined effect of restrictions on the transit system and government regulations that affect demand can substantially reduce the impact of an outbreak on society. The results underscore the importance of coordinated measures in managing public health risks during pandemics.

To determine changes in the number of infected passengers in a more refined way, we collected highly endangered passengers—those with a final infection probability greater than 50%—from the different demand and capacity-reduced scenarios. The table 3 provides a different view of the results.

**Table 3:** Percentage of highly endangered passengers across different scenarios (defined as passengers with a final infection probability exceeding 50%). In the scenario with full demand and no capacity restrictions, 24% of passengers are classified as highly endangered.

	90% capacity	80% capacity	70% capacity	50% capacity
100% demand	23.3%	23%	22.3%	20%
83% demand	22%	21.5%	20.8%%	18.3%
66.5% demand	19.5%	19%	18.6%	16%
59% demand	17.6%	17.3%	16.9%	14.8%
50% demand	16.3%	16%	15.6%	13.7%

In the original scenario, with full capacity and demand, capacity restrictions reduced the percentage of highly endangered individuals from 23.3% to 20%. The results follow the same trend as the global infection percentage: as demand decreases and capacity restrictions are introduced, this percentage declines further. For example, when capacity restrictions increase from 0% to 50%, the percentage drops to 16.6%, reducing the proportion of endangered passengers by 7%. Furthermore, when decision-makers implement 50% capacity restrictions along with local measures that reduce demand by 50%, the proportion of individuals at a critical infection level decreases to 13.7%.

The results highlight that demand-related restrictions have a stronger effect on the infection rate. Although capacity restrictions are essential parts of containment strategies, they are not necessarily enough to minimize the negative impact of an outbreak situation. Finally, combining transit system-related measures with demand-reducing decisions can maximize the reduction in infected individuals.

### Identifying critical components

Bota et al.^29^ proposed a methodology for identifying the most critical bus trips responsible for spreading disease among passengers. Here we follow the same approach by taking the average infection probabilities for all passengers on a particular vehicle trip to estimate the likelihood of transporting infected passengers. From another perspective, this gives us an estimate of the probability of getting infected if a passenger uses that specific vehicle trip. To calculate the most critical parts of the system, we take the top 100 vehicle trips based on this metric, according to the highest infection likelihoods.

Since we are interested in measuring the impact of the scenarios on the epidemiological risk associated public transportation system components, we took a slightly different approach. Instead of focusing on individual vehicle trips, we evaluated a similar likelihood of infection at the transit route level as a whole (i.e., any vehicle traveling on a particular route within a 24-hour time-frame). In the following part of the paper, we summarize the results for the top 10 most critical bus routes identified using this approach.

**Fig. 7:**
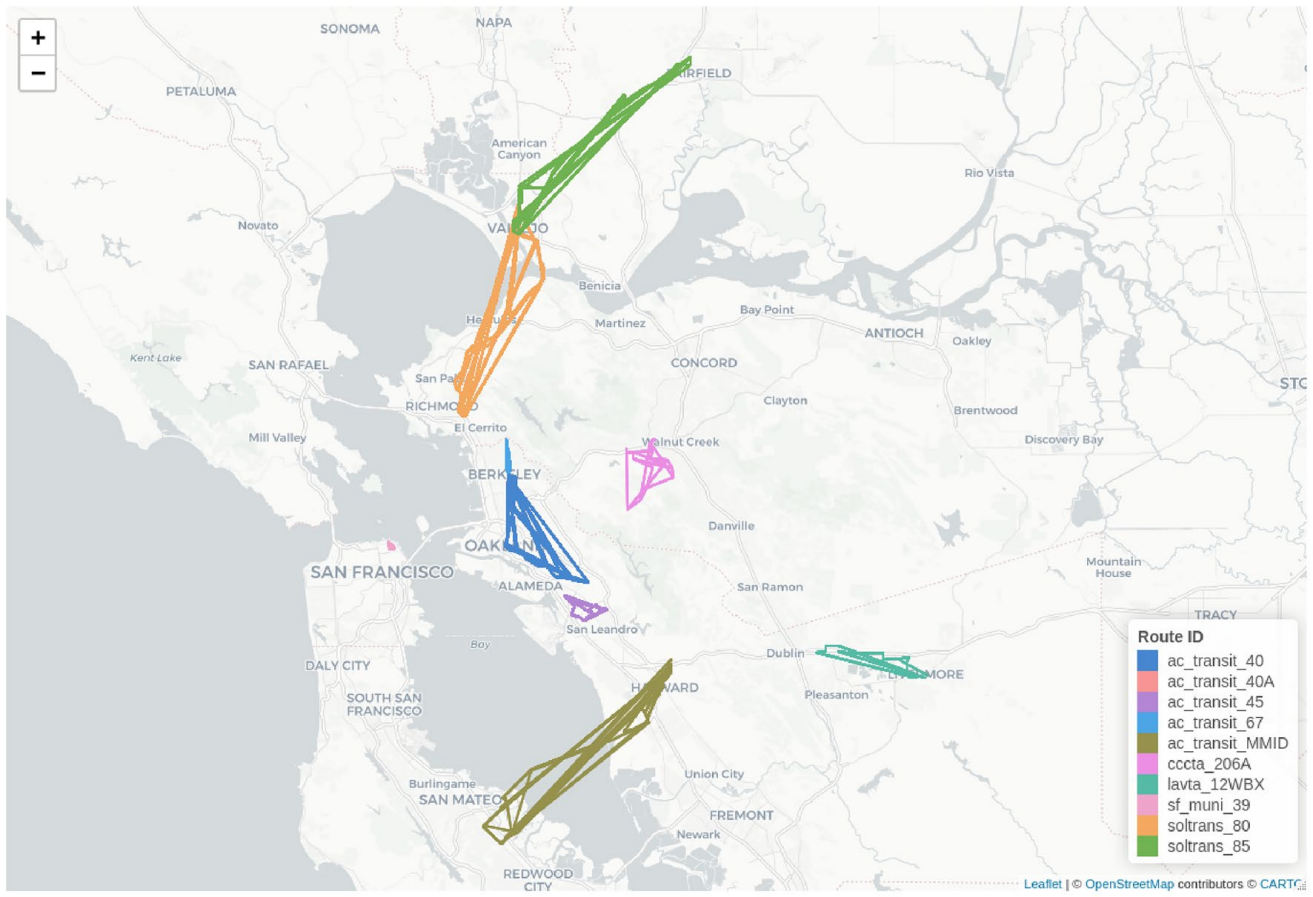
Critical regions in the transit system. Figure created with R version 4.1.2 https://cran.r-project.org/bin/windows/base/old/4.1.2/, using leaflet 2.2.2 https://cran.r-project.org/package=leaflet. The base tile layer was downloaded from OpenStreetMap (CC BY-SA 2.0; https://www.openstreetmap.org/copyright).

Figure 7 shows the most critical bus routes and the regions they connect. As shown, these transit routes are located in different parts of the Bay Area region, and most of them connect different counties or cities. However, there are other routes, for example, cccta_206A, operated by the Central Contra Costa Transit Authority, or sf_muni_39, which operates within San Francisco. The most critical routes include the connection between Fremont and Palo Alto, Richmond and Vallejo, Vallejo and Fairfield, or Berkeley and San Leandro. If passengers use these routes, the likelihood of getting infected is higher than 90% for baseline scenario. In the rest of this section, we evaluate how the different demand and vehicle capacity-related restrictions affect these routes.

**Fig. 8:**
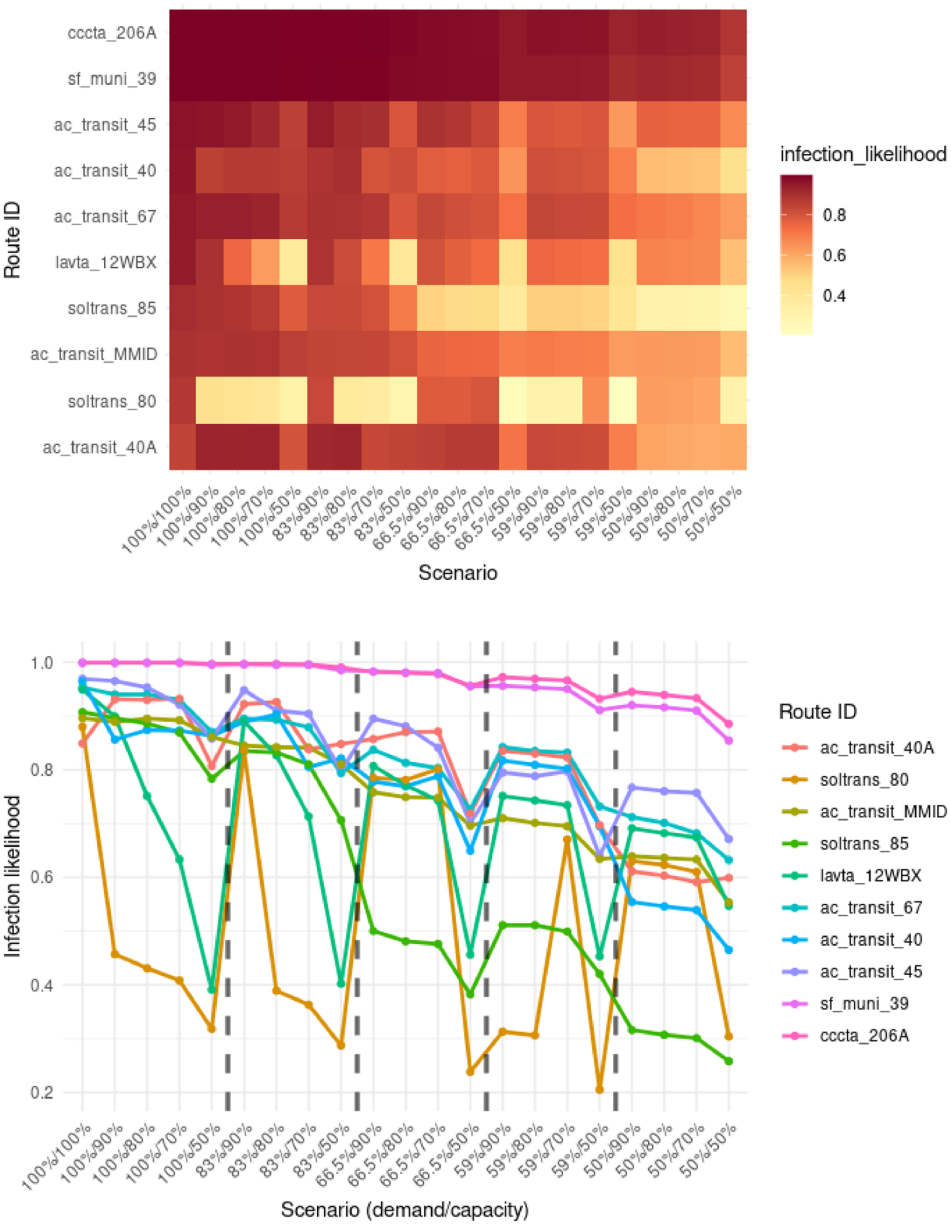
Effect of different scenarios for the top 10 most contagious vehicles. The x-axis shows the demand and capacity percentages of the different scenarios. Dashed lines separate scenario groups with differing levels of transit demand.

Figure 8 shows the changes in the expected likelihood of infection across varying scenarios with different vehicle capacities and reduction in travel demand. It can be seen that the probability of infection decreases with reduced transit demand for all identified routes. Among the top 10 routes, two types of patterns can be observed. The first pattern involves routes, such as in the case of sf_muni_39 or cccta_206A, where the likelihood of infection decreased slightly for the different scenarios. However, interesting trends can be observed in the rest of the routes, especially the soltrans_80 route that connects the cities of Fairfield and Vallejo, and lavta_12WBX operated by the Wheel public bus service in the Tri-Valley region. We can spot the drastic impact of vehicle capacities on the likelihood of infection for these routes. There is a 50–70% decrease in the probability of expected infection specifically due to restrictions in vehicle capacity. In some cases, demand-related changes did not impact the route-related risks. In other cases, these restrictions were effective in reducing disease-related dangers. Results show that some routes react differently to the various types of restrictions, highlighting the importance of combining multiple measures. This strategy will balance the reduction effect in the different parts of the system.

## Limitations

The primary limitation of the study is the size of the demand data used in the simulation environment. Although the GTFS structure is designed to serve hundreds of thousands on a daily basis, computational constraints and the transit model prevented us from simulating the real-world demand on the transit network. As a result, long train routes were not filled beyond half capacity, making it impossible to realistically simulate disease transmission on trains. Therefore, the results of this research focused on buses where the simulation was closer to a realistic scenario. It is also important to note that the study’s main aim was to demonstrate the effects of demand and vehicle capacity reductions by analyzing the differences between the baseline and reduced scenarios. While our proposed method serves as proof of concept, it can be directly applied to more comprehensive travel data sets in future research.

Furthermore, we acknowledge that the FAST-TrIPs assignment model used in our work seems to produce artifacts; specifically, it is unable to allocate a small percentage of public transit demand, even in the original, unaltered scenarios. We highlight however, that the FAST-TrIPs model has been used successfully in multiple studies^30,44,45^, and is generally considered to be a sound choice for public transit assignment.

## Conclusions

The results presented in this study show that restricting vehicle capacity impacts epidemiological spread, reducing the risk of transmission for travelers within the public transportation system. However, government policies aimed at reducing demand have a greater potential to decrease both the percentage of infected passengers and the number of highly infected individuals.

We demonstrated this by generating passenger contact networks for the public transit system in the San Fransisco Bay Area for multiple outbreak scenarios, corresponding to altered demand and capacity constraints. Simulated infection models were then applied to measure the spread of diseases on this network.

Our results indicate that reducing demand by 33.5% results in a drop in the average infection rate from 30.94% to 25.54%. Interestingly, we also found that the number of passengers highly susceptible to infection is even more sensitive to demand, with a potential decrease of up to 71% when demand is reduced. We also observed that solely relying on capacity restrictions risks the operability of the system, resulting in a greater number of passengers who cannot reach their destination using only the public transportation system. Depending on the level of demand, a 50% restriction in capacity may double the number of passengers unable to reach their destination.

According to our findings, we can conclude that combining demand-reducing government measures with transit system-related restrictions provides the best performance in reducing the expected number of infections, while maintaining the system’s ability to deliver passengers to their destinations. We acknowledge that due to computational limitations, we were unable to simulate the total number of passengers traveling on the public transportation system. We aim to extend our methodology to more realistic settings in the future.

## Data Availability

All input files are available on http://zenodo.org/records/15294444
